# COSIMO – patients with active cancer changing to rivaroxaban for the treatment and prevention of recurrent venous thromboembolism: a non-interventional study

**DOI:** 10.1186/s12959-018-0176-2

**Published:** 2018-09-04

**Authors:** Alexander T. Cohen, Anthony Maraveyas, Jan Beyer-Westendorf, Agnes Y. Y. Lee, Lorenzo G. Mantovani, Miriam Bach, on behalf of the COSIMO Investigators, Hayley Dawson

**Affiliations:** 1 Department of Haematological Medicine, Guys and St Thomas’ NHS Foundation Trust, King’s College London, Westminster Bridge Road, London, UK; 2 Joint Centre for Cancer Studies, Hull York Medical School, QCOH, Castle Hill Hospital, Hull, UK; 3 Thrombosis Research Unit, Department of Medicine I, Division Hematology, University Hospital “Carl Gustav Carus” Dresden, Dresden, Germany; 4 Kings Thrombosis Service, Department of Haematology, Kings College London, London, UK; 5 Division of Hematology, University of British Columbia, British Columbia Cancer Agency, Vancouver, BC Canada; 6 CESP-Center for Public Health Research, University of Milan Bicocca, Monza, Italy; 7 Bayer AG, Berlin, Germany

**Keywords:** Active cancer, Health-related quality of life, Low molecular weight heparin, Patient preference, Recurrent venous thromboembolism, Rivaroxaban, Vitamin K antagonist

## Abstract

**Background:**

Around 20% of venous thromboembolism (VTE) cases occur in patients with cancer. Current guidelines recommend low molecular weight heparin (LMWH) as the preferred anticoagulant for VTE treatment. However, some guidelines state that vitamin K antagonists (VKAs) and direct oral anticoagulants (DOACs) are acceptable alternatives for long-term therapy in some patients if LMWHs are not available. LMWHs and VKAs have a number of drawbacks that can increase the burden on patients. DOACs, such as rivaroxaban, can ameliorate some burdens and may offer an opportunity to increase patient satisfaction and health-related quality of life (HRQoL). The **C**ancer-associated thr**O**mbo**SI**s – patient-reported outco**M**es with rivar**O**xaban (COSIMO) study is designed to provide real-world information on treatment satisfaction in patients with active cancer who switch from LMWH or VKA to rivaroxaban for the treatment of acute VTE or to prevent recurrent VTE.

**Methods:**

COSIMO is a prospective, non-interventional, single-arm cohort study that aims to recruit 500 patients in Europe, Canada and Australia. Adults with active cancer who are switching to rivaroxaban having received LMWH/VKA for the treatment and secondary prevention of recurrent VTE for at least the previous 4 weeks are eligible. Patients will be followed for 6 months. The primary outcome is treatment satisfaction assessed as change in the Anti-Clot Treatment Scale (ACTS) Burdens score at week 4 after enrolment compared with baseline. Secondary outcomes include treatment preferences, measured using a discrete choice experiment, change in ACTS Burdens score at months 3 and 6, and change in HRQoL (assessed using the Functional Assessment of Chronic Illness Therapy – Fatigue questionnaire). COSIMO will collect data on patients’ medical history, patterns of anticoagulant use and incidence of bleeding and thromboembolic events. Study recruitment started in autumn 2016.

**Conclusions:**

COSIMO will provide information on outcomes associated with switching from LMWH or VKA therapy to rivaroxaban for the treatment or secondary prevention of cancer-associated thrombosis in a real-life setting. The key goal is to assess whether there is a change in patient-reported treatment satisfaction. In addition, COSIMO will facilitate the evaluation of the safety and effectiveness of rivaroxaban in preventing recurrent VTE in this patient population.

**Trial registration:**

NCT02742623. Registered 19 April 2016.

**Electronic supplementary material:**

The online version of this article (10.1186/s12959-018-0176-2) contains supplementary material, which is available to authorized users.

## Background

Cancer and its treatments (e.g. chemotherapeutic or anti-angiogenic agents) are well-established risk factors for venous thromboembolism (VTE) [1], and up to 20% of patients with active cancer will develop VTE, depending on the cancer type, stage and treatment [2, 3]. Cancer-associated thrombosis (CAT) has a significant impact on prognosis and patients’ quality of life (QoL). CAT is a leading cause of death among patients with cancer [4]; survivors of an initial event are at higher risk of recurrent events and bleeding during anticoagulation therapy compared with patients with VTE without malignancy [3, 5]. In the CLOT, CATCH and DALTECAN studies evaluating low molecular weight heparin (LMWH) therapy for the treatment of CAT, the residual risk of a recurrent event with 6 months’ LMWH therapy was ~7–9%, and that for major bleeding was ~2–6% [6–8]. CAT not only adds to the symptomatic burden of cancer but also to the treatment burden and emotional trauma caused by cancer and its treatment [9]. The risk of CAT is at its highest in the first few months after cancer diagnosis [10], and patients may already require multiple concurrent anti-neoplastic and supportive therapies during this time. Furthermore, the occurrence of CAT may delay critical treatments for cancer, including chemotherapy and surgery [11].

Current guidelines for the treatment of CAT recommend LMWH for initial and long-term (at least 3–6 months) therapy [12–15]. The American Society of Clinical Oncology (ASCO) guidelines also consider vitamin K antagonists (VKAs) as an acceptable alternative for long-term therapy if LMWHs are not available [16]. Although efficacious, both LMWH and VKAs have drawbacks that impose significant challenges in the care of patients with CAT: daily injections and a risk of heparin-induced thrombocytopenia with LMWHs, and frequent international normalised ratio monitoring and numerous food and drug interactions with VKAs [17].

Direct oral anticoagulants (DOACs; apixaban, dabigatran, edoxaban and rivaroxaban) were developed to overcome some of the limitations associated with traditional anticoagulants, and are now recommended over [15] or as an alternative [18] to LMWH/VKA therapy for long-term VTE treatment in patients without cancer. They have the potential benefits of fixed-dosing, no requirement for routine coagulation tests and few drug or food interactions, in addition to oral administration [17]. The phase III Hokusai-VTE-Cancer and select-d pilot trials provided the first randomised comparisons of edoxaban and rivaroxaban, respectively, versus dalteparin for the treatment of CAT, supporting their use in some patients [19, 20]. Recently published international guidance suggests that DOACs can be considered for treatment of CAT in patients with stable cancer not receiving systemic anti-cancer therapy, and in cases where a VKA is an acceptable treatment choice [21]; this is also reflected in the most recent American College of Chest Physicians guidelines update [15].

**Fig. 1:**
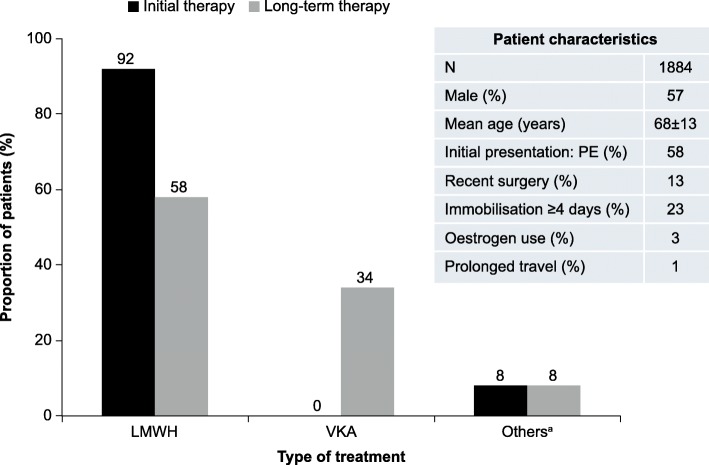
Initial and long-term anticoagulant therapy in patients with cancer^b^ and a first episode of VTE – data from the RIETE registry [22]. ^a^Includes unfractionated heparin and thrombolytic agents. ^b^Defined as newly diagnosed cancer, metastatic cancer or cancer undergoing treatment. LMWH, low molecular weight heparin; PE, pulmonary embolism; VKA, vitamin K antagonist; VTE, venous thromboembolism

The burden of care associated with traditional anticoagulation therapies for CAT may explain the high levels of non-adherence to current guidelines and frequent switching between anticoagulation therapies in clinical practice. In Europe, over 90% of patients receiving treatment for active cancer and first VTE are initially prescribed LMWH for the prevention of VTE recurrence; approximately 30% are subsequently switched to VKAs for long-term therapy (Fig. 1) [22]. In a retrospective analysis of 52,911 patients with CAT from the US MarketScan Treatment Pathways database, 50% of patients were initially prescribed warfarin [23] despite guidelines recommending LMWH [16]. Furthermore, of the 40% of patients initially prescribed LMWH, 44% switched to another anticoagulant within 1 month [23]. Patient involvement and treatment satisfaction are increasingly emphasised as key to improving adherence with long-term therapy [24, 25]. Unfortunately, there is only limited real-world information on patient satisfaction with or preferences for different anticoagulants for CAT treatment [25].

This paper presents the study design and rationale for the Cancer-associated thrOmboSIs – patient-reported outcoMes with rivarOxaban (COSIMO) study, which aims to collect prospective real-world data on patient satisfaction with anticoagulation treatment after a switch from LMWH or VKA to rivaroxaban in patients with cancer. In addition, COSIMO will facilitate the evaluation of adverse events (AEs) and the recurrence of VTE with rivaroxaban in this patient population. COSIMO is part of the Cancer Associated thrombosis – expLoring soLutions for patients through Treatment and Prevention with RivarOxaban (CALLISTO) programme (Table 1) [20].

**Table 1:** Studies included in the CALLISTO programme^a^

Study name	Study designand patient population	Dose	Clinical trial reference	Status
VTE prevention				
CASSINI	Prospective, randomised, double-blind, placebo-controlled superiority analysis in patients at high risk of VTE due to initiate chemotherapy for cancer	Rivaroxaban 10 mg od for 6 months	NCT02555878	Ongoing
PRO-LAPS 2 IIR	Randomised, double-blind, placebo-controlled study of extended antithrombotic prophylaxis in patients after laparoscopic surgery for colorectal cancer	Rivaroxaban 10 mg od for 28 days with 2 months of follow-up	NCT03055026	Ongoing
VTE treatment				
select-d IIR	Randomised, open-label, multicentre pilot study, with a second placebo-controlled randomisation, comparing the duration of anticoagulation therapy (6 months vs 12 months) in adult patients with residual vein thrombosis	Dalteparin (200 IU/kg od for the first 30 days, followed by 150 IU/kg od) Rivaroxaban (15 mg bid for 21 days, followed by 20 mg od)	EudraCT 2012-005589-37	Results reported [20]
CASTA-DIVA IIR	Randomised, open-label pilot study in patients with active cancer and confirmed acute VTE	Dalteparin 200 IU/kg od for 4 weeks, followed by 150 IU/kg od for 8 weeks Rivaroxaban 15 mg bid for 3 weeks, followed by 20 mg od for 9 weeks	NCT02746185	Ongoing
CONKO-011 IIR	Prospective, randomised, open-label, multicentre study in patients with active cancer and confirmed acute VTE	LMWH as per label for 3 months Rivaroxaban 15 mg bid for 21 days, followed by 20 mg od for 3 months	NCT02583191	Ongoing
Investigator- initiated quality assessment initiative	Follow-up of 200 patients with cancer-associated thrombosis who previously received rivaroxaban for 6 months	Rivaroxaban 15 mg bid for 3 weeks, then 20 mg od (reduced in patients aged >75 years)	N/A	Results reported [36]
COSIMO	Patient-reported outcomes, follow-up for 6 months	Rivaroxaban as per label	NCT02742623	Ongoing
FRONTLINE 2 survey^c^	Second non-interventional study of current practice in the treatment of cancer-associated thrombosis. Up to 5000 oncologists and haematologists will be surveyed	N/A	N/A	Ongoing

^a^Please see https://www.xarelto.com/en/resources/newsfeed/bayer-extends-clinical-investigation-of-xarelto-for-the-prevention-and-treatment-of-life-threatening-blood-clots-in-patients-with-cancer/ (accessed 19 Jun. 2018) for information about the CALLISTO programme. ^b^Please see http://frontline2.tri-london.ac.uk/ for information on the FRONTLINE 2 survey

*bid*, twice daily; *IIR* investigator-initiated research, *LMWH* low molecular weight heparin, *N/A* not applicable, *od* once daily, *VTE* venous thromboembolism

## Methods

### Study design and patient population

COSIMO is a prospective, non-interventional, single-arm cohort study that is recruiting patients at approximately 70 sites across Australia, Belgium, Canada, Denmark, France, Germany, Italy, Netherlands, Spain and the UK.

Adults with active cancer and acute deep vein thrombosis (DVT) and/or pulmonary embolism (PE), or with recurrent VTE, who are scheduled to be switched to rivaroxaban after having received standard of care (SOC) anticoagulation therapy (either LMWH or a VKA) for CAT for ≥4 weeks are eligible. Patients with an Eastern Cooperative Oncology Group (ECOG) performance status score of 0, 1 or 2 will be included. ‘Active cancer’ includes cancer (other than fully treated basal cell or squamous cell carcinoma of the skin) that has been diagnosed or treated within the previous 6 months, or recurrent or metastatic cancer. Inclusion and exclusion criteria are shown in Table 2.

**Fig. 2:**
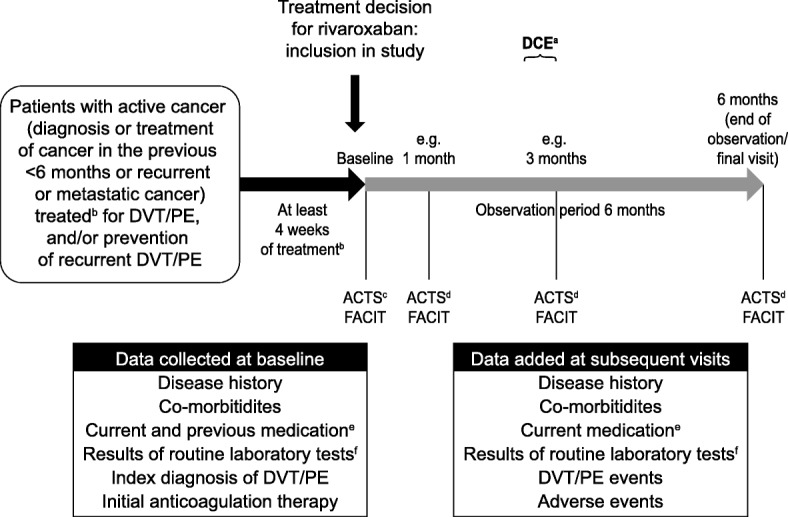
COSIMO – study design and data collection. ^a^DCE per telephone interview 4–12 weeks after starting rivaroxaban treatment. ^b^Patients treated for at least 4 weeks of SOC anticoagulation therapy with LMWH or VKA therapy. ^c^For previous anticoagulation therapy. ^d^For rivaroxaban treatment. ^e^Including anti-cancer medication. ^f^Haemoglobin, haematocrit, white blood cells, platelets, electrolytes, C-reactive protein, serum creatinine, CrCl, liver enzymes and haemoccult test. ACTS, Anti-Clot Treatment Scale; CrCl, creatinine clearance; DCE, discrete choice experiment; DVT, deep vein thrombosis; FACIT, Functional Assessment of Chronic Illness Therapy – Fatigue questionnaire; LMWH, low molecular weight heparin; PE, pulmonary embolism; SOC, standard of care; VKA, vitamin K antagonist

Patients enrolled into the study will be observed for 6 months. Treatment duration is at the physician’s discretion and is not dependent on the initially scheduled treatment duration. In addition to contact at enrolment and the end of the 6-month observational period, patients should undergo two follow-up visits (at approximately week 4 and month 3; timepoints of interest for data collection) (Fig. 2). Owing to the observational nature of the study, the protocol does not define the exact dates for the two follow-up visits, and investigators are advised to schedule these to coincide with regular physician appointments.

### Study outcomes

The primary outcome of the COSIMO study is treatment satisfaction, assessed as change in the Anti-Clot Treatment Scale (ACTS) Burdens score [26] from enrolment to week 4. Secondary outcomes include patient preferences with regard to convenience attributes; the change in ACTS Burdens score at month 3 and month 6; change in health-related QoL (HRQoL); patterns of anticoagulant use and incidence of bleeding, thromboembolic events and other AEs and serious AEs.

### Data collection and management

Data collection is illustrated in Fig. 2. Treatment-related data will be collected at baseline and during visits that take place in routine clinical practice. Data will be recorded in an electronic case report form. The information collected at enrolment will include prior medical history and current co-morbidities, current and previous medication, a description of the index venous thromboembolic event and its treatment, and the results of routine laboratory tests. The reasons for switching to rivaroxaban, planned treatment duration and dose will also be recorded.

Treatment satisfaction will be measured using the self-administered ACTS questionnaire. Patients will be asked to complete the questionnaire at enrolment and, after the initiation of rivaroxaban therapy, at approximately week 4, month 3 and month 6 (end of the observation period). During this time, the investigator may decide to change anticoagulation therapy; in these circumstances, the patient would remain in the study until the end of the 6-month follow-up period but would not need to complete any further ACTS questionnaires. The Functional Assessment of Chronic Illness Therapy (FACIT) Fatigue questionnaire will be used to assess HRQoL and will be completed alongside the ACTS questionnaire at enrolment, during the two follow-up visits and at the end of the observation period. Information on convenience-related patient preferences in anticoagulation treatment will be collected by means of a discrete choice experiment (DCE) in a semi-structured telephone interview. Patients will be asked to volunteer to take part in the DCE, which will be conducted by telephone at 4–12 weeks after enrolment.

AEs and serious AEs will be documented up to the completion of the 6-month observation period or up to 30 days after rivaroxaban discontinuation, whichever occurs earlier. Bleeding events (collected as serious AEs or non-serious AEs) will be adjudicated and categorised as major or non-major bleeding. Thromboembolic events, including incidental thromboembolic events documented in routine imaging (e.g. incidental PE from staging computed tomography; collected as serious AEs or non-serious AEs) will be adjudicated and categorised (symptomatic or incidental). An independent Central Adjudication Committee of four expert physicians will adjudicate major bleeding and thromboembolic events (recurrent VTE, other thromboembolic events, major adverse cardiovascular events). All events resulting in death (as reported by the investigator) will be adjudicated. Causes of death will be categorised as being related to cancer, thrombosis, bleeding, infectious diseases or other.

In cases of rivaroxaban discontinuation, the reason for permanent cessation and potential switch to another anticoagulant will be documented.

### Study questionnaires

The ACTS questionnaire uses a five-point Likert scale, ranging from ‘5 = not at all’ to ‘1 = extremely’, to assess patient response [26] (Additional file 1). It is a self-administered instrument that includes 13 items about the burden of anticoagulant therapy (bleeding, bruising, limitation of activities, food and drink limitations, need to avoid other medications, daily inconveniences, occasional inconveniences, adherence issues, time spent on regimen, anxiety, frustration and overall burden), and four items about the benefits of anticoagulant therapy (confidence, reassurance, satisfaction and overall benefit) [26]. The use of separate subscales for ACTS Burdens and Benefits means that it will be possible to focus specifically on the burdens of anticoagulant therapy as the primary outcome [26]. Because the ACTS questionnaire has a recall period of 4 weeks, data should be collected between -2 to +4 weeks around each visit. Further information is given in Additional file 1.

During the DCE, participants will be asked to make a choice between options ‘A’ and ‘B’ across nine treatment scenarios (plus a control scenario) on pictorial charts, considering differing combinations of utility-increasing and utility-decreasing attribute levels (trade-off type of choice) [27]. The aim of the DCE is to define the ideal anticoagulant treatment from the perspective of patients with CAT (Additional file 2).

FACIT Fatigue is a 13-item questionnaire that assesses feelings of tiredness, weakness, listlessness, frustration, energy levels, ability to perform daily tasks (including eating) and need for help to complete tasks [28]. Patients will score each item on a five-point (0 to 4) scale; a higher score indicates better HRQoL (Additional file 3).

### Sample size calculation

The sample size calculation was based on the primary outcome, a change in ACTS Burdens score at week 4 after enrolment compared with baseline. Based on data from the XALIA cancer subgroup analysis (data on file) [29], the mean difference in ACTS Burdens score between enrolment and week 4 was assumed to be 1.3, with a standard deviation of 8.0 considered reasonable. Based on these assumptions, 300 patients will be needed to reach a power of 80% for the primary analyses. Considering high drop-out rates in this patient population [6], 375 patients should be included to ensure sufficient numbers for the primary analyses. Given the heterogeneous nature of the cancer population and expected high drop-out rates after week 4, the COSIMO study aims to enrol 500 patients overall to have sufficient numbers for the secondary analyses.

### Statistical analyses

Analyses will generally include all patients who received at least one dose of rivaroxaban, and who completed the ACTS questionnaire at the particular time point being assessed (e.g. week 4, month 3 or month 6). The questionnaire responses are multiple measurements on patient-reported treatment satisfaction over time; therefore, a mixed model repeated measures analysis will be used to analyse the data. The null hypothesis is no change in ACTS Burdens score between enrolment and week 4; hypothesis testing will be at a 5% significance level. The change in the ACTS Burdens score is assumed to be normally distributed and will be analysed using a paired t-test. The assumption of normality will be tested using the Shapiro–Wilk test at the 0.10 level of significance. If the test shows significance, the Wilcoxon signed-rank test will be used. For missing items, imputation to the mean will be used where there are >50% of the questions (>6 items for ACTS Burdens) completed. Otherwise, the item will be regarded as a missing value. Subgroup analyses, by type and duration of SOC therapy and by reason for switching from SOC, will be provided. Sensitivity analyses will be conducted to investigate the potential impact of patients discontinuing the study earlier than week 4 on the outcome.

## Discussion

For some patients undergoing treatment for cancer, the necessity for anticoagulant therapy may be regarded as an added burden [30]. LMWHs are recommended as first-line therapy for acute and long-term treatment of CAT in clinical guidelines; nevertheless, many patients with CAT are switched to, or even initiated on, a VKA [22, 23], possibly because of a preference for oral over injectable therapies [25] or for cost reasons. DOACs such as rivaroxaban are considered more convenient than VKAs because of their simple dosing regimens and lack of the need for routine coagulation monitoring [31, 32]. A subgroup analysis of pooled results from EINSTEIN DVT and EINSTEIN PE demonstrated that the rate of recurrent VTE was similarly reduced in patients treated with rivaroxaban or enoxaparin/VKA therapy, and that the number of major bleeding events was reduced with rivaroxaban therapy in patients with or without active cancer [33]. More recently, the efficacy and safety of edoxaban and rivaroxaban for the treatment of CAT have been demonstrated in the first randomised head-to-head comparisons with a LMWH (dalteparin) in the phase III Hokusai-VTE-Cancer study [34] and the phase III pilot study select-d [20], respectively. Several studies on the use of rivaroxaban for the treatment of CAT in clinical practice have also been published; these results provide some reassurance that rivaroxaban is safe and effective in this clinical setting [29, 35–37]. Furthermore, in the EINSTEIN studies, patients treated with rivaroxaban reported greater treatment satisfaction than patients treated with enoxaparin/VKA, as measured by the ACTS questionnaire [32, 38]. The role of DOACs in the treatment and secondary prevention of CAT is being investigated in ongoing studies [39–43].

The COSIMO study aims to collect real-world data in consecutive patients with cancer switching from SOC therapy (LMWH or VKA) to rivaroxaban in circumstances where SOC therapy cannot be continued [44]. The study began recruiting patients in October 2016. The primary outcome is a change in patient-reported treatment satisfaction (specifically, the ACTS Burdens score) between baseline (the point of switching) and week 4. Treatment satisfaction will also be measured at month 3 and at the end of the 6-month observation period, so that changes in ACTS Burdens and Benefits scores can be compared over time. Effectiveness and safety data will be gathered through AE reporting by study investigators. To improve the current understanding of treatment needs, comprehensive data on cancer type and stage, treatment patterns and clinical management will be collected. In this regard, COSIMO will provide prospective real-world data on the effectiveness and safety of rivaroxaban according to cancer type and stage, as well as on overlapping toxicities and interactions between rivaroxaban and anti-cancer therapies. COSIMO will also contribute important information on the management of challenging patient populations with CAT, such as patients in whom AEs occur because of chemotherapeutic agents (e.g. thrombocytopenia) or patients who require surgery or other interventions (biopsies, etc.).

**Table 2:** Inclusion and exclusion criteria for the COSIMO study

Inclusion criteria	Exclusion criteria
Adult female and male patients with active cancer other than fully treated basal-cell or squamous-cell carcinoma of the skin (active cancer defined as the diagnosis or treatment of cancer in the previous <6 months or recurrent or metastatic cancer)	Contraindicated for rivaroxaban
Patients who have been treated with SOC anticoagulation (LMWH/VKA) for treatment of DVT and/or PE (index venous thromboembolic event), and/or prevention of recurrent DVT and PE for ≥4 weeks prior to inclusion in the study	Experienced an index VTE despite chronic anticoagulant therapy
Decision taken to start rivaroxaban for the treatment of DVT and/or PE and/or the prevention of recurrent DVT and/or PE	Receiving apixaban, edoxaban or dabigatran or any investigational drug as initial therapy for index VTE
ECOG performance status score of 0, 1 or 2	Participating in a clinical study using investigational drugs^a^
Provided informed consent	
Available for follow-up with a life expectancy >6 months	

^a^Except as part of an investigational oncology trial

*DVT* deep vein thrombosis, *ECOG* Eastern Cooperative Oncology Group, *LMWH* low molecular weight heparin, *PE* pulmonary embolism, *SOC* standard of care, *VKA* vitamin K antagonist, *VTE* venous thromboembolism

COSIMO is a non-interventional study; therefore, the inclusion and exclusion criteria are deliberately minimal, to mirror real-world practice. However, there are some restrictions to enrolment to ensure the following: the welfare of the population under study (e.g. inclusion is restricted to patients with an ECOG performance status score of ≤2) and alignment with current guideline recommendations (e.g. exclusion of patients pre-treated with anticoagulants other than SOC). These criteria will also ensure a level of study homogeneity for the facilitation of data analyses.

The choice of instruments for evaluating HRQoL is critical for accurate interpretation of patient self-reporting. The COSIMO study will use the ACTS and a DCE to record patient treatment satisfaction and preferences, respectively, and FACIT Fatigue instruments to measure changes in HRQoL relating to the cancer itself.

The ACTS questionnaire is specific for anticoagulation and, therefore, the score should not be affected by the patient’s cancer stage and/or cancer treatment. It is a modified form of the Duke Anticoagulation Satisfaction Scale, a 25-item, single scale, which is used to assess limitations, inconveniences and/or discomforts, as well as positive impacts, related to anticoagulant treatment [45]. ACTS was validated using data from the EINSTEIN DVT study [31], which included patients with acute symptomatic DVT treated with rivaroxaban or enoxaparin/VKAs [46]. A rigorous development process was used to ensure that it was appropriate for patients with atrial fibrillation and VTE globally [31]. The DCE is a validated tool for assessing patient preference for anticoagulation therapy [47–49].

Fatigue is one of the most common side effects in patients with cancer who are receiving cancer therapy [28, 50], and it may have a pervasive effect on treatment satisfaction. The FACIT Measurement System offers several benefits for measuring HRQoL in people with cancer and other chronic diseases and has proven utility for measuring change in HRQoL in observational studies [51, 52]. The content was developed jointly by experts and patients, and the scales have been validated in patients with different forms of cancer [52].

One of the limitations of this study is that it was not designed to examine the impact of cancer subtypes, or other potential confounding factors that vary over time, on outcomes. An additional limitation, which applies to all studies enrolling patients with cancer, is the high discontinuation rates over time. Nonetheless, this should have minimal impact on the primary outcome, which is measured at week 4 after initiation of treatment with rivaroxaban; there would likely be minimum impact on other outcomes. There might also be the potential to overestimate treatment satisfaction with rivaroxaban due to selection bias, because the patients eligible for COSIMO (or their physicians) had chosen not to continue with SOC treatment. Finally, the lack of a control patient cohort might make it difficult to put the results into perspective, but finding a matched comparator group of patients with CAT would have been a major challenge.

## Conclusions

The ongoing COSIMO study is designed to evaluate satisfaction with anticoagulation treatment in patients with active cancer who are at risk of recurrent VTE or have switched from LMWH/VKA to rivaroxaban. It will also evaluate the safety and effectiveness of rivaroxaban in preventing recurrent VTE in this important patient population. The evaluation tools in this study – the ACTS and FACIT Fatigue questionnaires and a DCE focused on treatment preferences – have been specifically chosen to provide information that might help guide the future management and treatment of patients coping with serious concurrent illnesses.

## Additional files

Additional file 1: Anti-Clot Treatment Scale (ACTS). (DOCX 30 kb)

Additional file 2: Discrete Choice Experiment (DCE). (DOCX 26 kb)

Additional file 3: Functional Assessment of Chronic Illness Therapy (FACIT) Fatigue score. (DOCX 27 kb)

## Abbreviations

ACTS: Anti-Clot Treatment Scale
AE: Adverse event
CALLISTO: Cancer Associated thrombosis – expLoring soLutions for patients through Treatment and Prevention with RivarOxaban
CAT: Cancer-associated thrombosis
COSIMO: **C**ancer-associated thr**O**mbo**S**Is – patient-reported outco**M**es with rivar**O**xaban
DCE: Discrete choice experiment
DOAC: Direct oral anticoagulant
DVT: Deep vein thrombosis
ECOG: Eastern Cooperative Oncology Group
FACIT: Functional Assessment of Chronic Illness Therapy
HRQoL: Health-related quality of life
LMWH: Low molecular weight heparin
PE: Pulmonary embolism
QoL: Quality of life
SOC: Standard of care
VKA: Vitamin K antagonist
VTE: Venous thromboembolism
