# Supplementary material for: COSIMO – patients with active cancer changing to rivaroxaban for the treatment and prevention of recurrent venous thromboembolism: a non-interventional study
Source: Thromb J. 2018 Sep 4;16:21. doi: 10.1186/s12959-018-0176-2 (PMC6122180; doi:10.1186/s12959-018-0176-2)
Supplement: Supplementary file 1 — Anti-Clot Treatment Scale (ACTS). (DOCX 30 kb) [file 12959_2018_176_MOESM1_ESM.docx]

Additional File 1: Anti-Clot Treatment Scale (ACTS)

This is a self-administered questionnaire designed to assess patient views associated with long-term use of anticoagulant therapies, irrespective of the underlying condition [1]. It is a modified form of the Duke Anticoagulation Satisfaction Scale [2], and was developed using data from the EINSTEIN DVT study [3]. Responders use a five-point Likert scale (see table below). When used in clinical research, it is recommended that the ACTS Burdens scores are reverse-scored (‘5 = extremely’ to ‘0 = not at all’) so that higher ACTS scores indicate greater satisfaction with treatment [1]; however, this was not done in this study.

In situations where an item has a missing score or where an item has been given more than one score, imputation to the mean can be used if >50% of the questions have responses. For the Burdens scale, the mean of the scores for completed answers should be multiplied by 12 to yield the overall score; for the Benefits scale, the mean of the scores for completed answers should be multiplied by four to yield the overall score. If <50% of the items have responses, the scale should be regarded as a missing value.

It is recommended that the questionnaire is completed by the respondent in the presence of a researcher. Patients should be given some information about how to complete the questionnaire, but if the patient has difficulty with self-completion then it may be administered as an interview in which the questions are read out loud at a slow pace.

**Table** Anti-Clot Treatment Scale [1]

| **During the past 4 weeks** | **Not at all** | **A little** | **Moderately** | **Quite a bit** | **Extremely** |
| --- | --- | --- | --- | --- | --- |
| 1. How much does the possibility of bleeding as a result of your anti-clot treatment limit you from taking part in vigorous physical activities? (e.g. exercise, sports, dancing etc.) | 1 | 2 | 3 | 4 | 5 |
| 1. How much does the possibility of bleeding as a result of your anti-clot treatment limit you from taking part in your usual activities? (e.g. work, shopping, housework etc.) | 1 | 2 | 3 | 4 | 5 |
| 1. How bothered are you by the possibility of bruising as a result of your anti-clot treatment? | 1 | 2 | 3 | 4 | 5 |
| 1. How bothered are you by having to avoid other medicines (e.g. aspirin) as a result of your anti-clot treatment? | 1 | 2 | 3 | 4 | 5 |
| 1. How much does your anti-clot treatment limit what you eat and drink (including alcohol)? | 1 | 2 | 3 | 4 | 5 |
| 1. How much of a hassle (inconvenience) are the daily aspects of your anti-clot treatment? (e.g. remembering to take your medicine at a certain time, taking the correct dose of your medicine, limiting what you eat and drink [including alcohol] etc.) | 1 | 2 | 3 | 4 | 5 |
| 1. How much of a hassle (inconvenience) are the occasional aspects of anti-clot treatment? (e.g. the need for blood tests, going to or contacting the clinic/doctor, making arrangements for treatment while travelling etc.) | 1 | 2 | 3 | 4 | 5 |
| 1. How difficult is it to follow your anti-clot treatment? | 1 | 2 | 3 | 4 | 5 |
| 1. How time-consuming is your anti-clot treatment? | 1 | 2 | 3 | 4 | 5 |
| 1. How much do you worry about your anti-clot treatment? | 1 | 2 | 3 | 4 | 5 |
| 1. How frustrating is your anti-clot treatment? | 1 | 2 | 3 | 4 | 5 |
| 1. How much of a burden is your anti-clot treatment? | 1 | 2 | 3 | 4 | 5 |
| 1. Overall, how much of a negative impact has your anti-clot treatment had on your life? | 1 | 2 | 3 | 4 | 5 |
| 1. How confident are you that your anti-clot treatment will protect your health? (e.g. prevent blood clots, stroke, heart attack, DVT, embolism) | 1 | 2 | 3 | 4 | 5 |
| 1. How reassured do you feel because of your anti-clot treatment? | 1 | 2 | 3 | 4 | 5 |
| 1. How satisfied are you with your anti-clot treatment? | 1 | 2 | 3 | 4 | 5 |
| 1. Overall, how much of a positive impact has your anti-clot treatment had on your life? | 1 | 2 | 3 | 4 | 5 |

ACTS Burdens: questions 1–13; ACTS Benefits questions 14–17. The global questions are those that start with ‘overall’ and are designed to measure the overall impact of treatment (both positive and negative), and are used to validate results from the ACTS Burdens- and ACTS Benefits-related questions.
ACTS, Anti-Clot Treatment Scale; DVT, deep vein thrombosis.

**References**

1. Cano SJ, Lamping DL, Bamber L, Smith S. The Anti-Clot Treatment Scale (ACTS) in clinical trials: cross-cultural validation in venous thromboembolism patients. Health Qual Life Outcomes. 2012;10:120.

2. Samsa G, Matchar DB, Dolor RJ, Wiklund I, Hedner E, Wygant G, et al. A new instrument for measuring anticoagulation-related quality of life: development and preliminary validation. Health Qual Life Outcomes. 2004;2:22.

3. The EINSTEIN Investigators. Oral rivaroxaban for symptomatic venous thromboembolism. N Engl J Med. 2010;363:2499-510.
