# Supplementary material for: COSIMO – patients with active cancer changing to rivaroxaban for the treatment and prevention of recurrent venous thromboembolism: a non-interventional study
Source: Thromb J. 2018 Sep 4;16:21. doi: 10.1186/s12959-018-0176-2 (PMC6122180; doi:10.1186/s12959-018-0176-2)
Supplement: Supplementary file 2 — Discrete Choice Experiment (DCE). (DOCX 26 kb) [file 12959_2018_176_MOESM2_ESM.docx]

Additional File 2: Discrete Choice Experiment (DCE)

A DCE is an attribute-based measure of benefit [1, 2]. Based on previous literature [3] and treatment characteristics, attributes identified as being important for this study are route of administration, frequency of dosing, the need for regular international normalised ratio monitoring, interactions with food/alcohol and distance to the patient’s treating physician. Note that the last attribute is the chosen neutral comparator attribute. It offers the possibility to express patient preferences with regard to the other four attributes and their levels in units of distance to the treating physician. Each of the attributes has two possible responses, A or B. This would result in 32 (2^5^) possible treatment combinations; for practical purposes, this was reduced to nine scenarios using the IBM SPSS statistics package (IBM; New York, NY). Charts summarising these scenarios in pictorial form have been prepared and will be sent to participating patients ahead of semi-structured telephone interviews, which will be conducted between week 4 and week 12 after enrolment. One additional chart (Chart 10 – a duplicate of one of the test charts in which A and B have been switched) will be used as a test scenario.

Patients participating in the DCE who have inconsistent (based on the results of the test scenario) or incomplete data will be excluded from the analysis data set. Generally, there will be no imputation of missing data. Patient choices will be analysed using descriptive statistics (as frequency of chosen attribute levels). Ultimately, the relative importance of each attribute for the overall decision for/against an option in the DCE will be calculated for the whole population and for population subgroups.

**References**

1. Reed Johnson F, Lancsar E, Marshall D, Kilambi V, Muhlbacher A, Regier DA, et al. Constructing experimental designs for discrete-choice experiments: report of the ISPOR Conjoint Analysis Experimental Design Good Research Practices Task Force. Value Health. 2013;16:3-13.

2. Ryan M. Discrete choice experiments in health care. BMJ. 2004;328:360-1.

3. Moia M, Mantovani LG, Carpenedo M, Scalone L, Monzini MS, Cesana G, et al. Patient preferences and willingness to pay for different options of anticoagulant therapy. Intern Emerg Med. 2013;8:237-43.
