# Supplementary material for: COSIMO – patients with active cancer changing to rivaroxaban for the treatment and prevention of recurrent venous thromboembolism: a non-interventional study
Source: Thromb J. 2018 Sep 4;16:21. doi: 10.1186/s12959-018-0176-2 (PMC6122180; doi:10.1186/s12959-018-0176-2)
Supplement: Supplementary file 3 — Functional Assessment of Chronic Illness Therapy (FACIT) Fatigue score. (DOCX 27 kb) [file 12959_2018_176_MOESM3_ESM.docx]

Additional File 3. Functional Assessment of Chronic Illness Therapy (FACIT) Fatigue score

The FACIT management system is a collection of nearly 60 quality-of-life questionnaires targeted at the management of specific types of cancer as well as treatment- and symptom-specific measures of quality of life in patients with cancer ([www.facit.org](http://www.facit.org/)).

FACIT Fatigue is a 13-item questionnaire that assesses feelings of tiredness, weakness, listlessness, frustration, energy levels, ability to perform daily tasks (including eating) and need for help to complete tasks. The items are scored on a five-point scale; the higher the overall score, the higher the patient’s quality of life [1]. Some items are scored from 0 to 4 where ‘0 = not at all’ and ‘4 = very much’ and others are reversed scored (‘0 = very much’ to ‘4 = not at all’). It was chosen for the COSIMO study because fatigue is one of the most common side-effects in patients undergoing treatment for cancer [2].

As long as more than 50% of the items have a response, values for missing responses can be calculated via prorating: the sum of the scores for the completed items (i.e. those with responses) is multiplied by 13 and then divided by the number of completed items. Patients will complete this instrument at enrolment and, for example, at week 4 and months 3 and 6; summary scores will be presented for each visit.

**References**

1. Kapoor A, Singhal MK, Bagri PK, Narayan S, Beniwal S, Kumar HS. Cancer related fatigue: A ubiquitous problem yet so under reported, under recognized and under treated. South Asian J Cancer. 2015;4:21-3.

2. Cella D, Peterman A, Passik S, Jacobsen P, Breitbart W. Progress toward guidelines for the management of fatigue. Oncology (Williston Park ). 1998;12:369-77.
